# Designing an ideal alcohol-based hand sanitizer: in vitro antibacterial responses of ethanol and isopropyl alcohol solutions to changing composition

**DOI:** 10.1186/s41120-021-00038-x

**Published:** 2021-11-22

**Authors:** Ifeanyi T. Nzekwe, Onyedika I. Agwuka, Moses U. Okezie, Daniel O. Fasheun, Petra O. Nnamani, Chukwuma O. Agubata

**Affiliations:** 1 Department of Pharmaceutics & Pharmaceutical Technology, Nnamdi Azikiwe University, Awka, Anambra Nigeria; 2 Department of Pharmaceutical Microbiology & Biotechnology, Nnamdi Azikiwe University, Awka, Anambra Nigeria; 3 Department of Biochemistry, Institute of Chemistry, Federal University of Rio de Janeiro, Rio de Janeiro, Brazil; 4 Department of Pharmaceutics, University of Nigeria, Nsukka, Enugu Nigeria; 5 Department of Pharmaceutical Technology and Industrial Pharmacy, University of Nigeria, Nsukka, Enugu State Nigeria

**Keywords:** Alcohol, Sanitizer, Disinfection, Stability, Additives

## Abstract

This study aimed to achieve an in vitro quantification of the effects of composition and formulation factors on the killing rates of alcohol-based hand sanitizers. The killing rates of 85% ethyl alcohol (ET) and isopropyl alcohol (IPA) were studied under different conditions such as pH, electrolyte concentration, or inclusion of herbal extracts (cucumber, carrot, and aloe vera), a quaternary ammonium compound, or thickener over different time intervals. Changes in the activities were retested after 3 months as an indication of stability. From two-way ANOVA, both the time of exposure and the sanitizer type affected the activity against *Staphylococcus aureus* (*P* = 0.001 for both alcohols), whereas for *Escherichia coli*, time of exposure was significant (*P* = 0.027), while sanitizer type was less significant (*P* = 0.063)*.* Extreme pHs, the presence of ions, and the inclusion of additives such as benzalkonium chloride (BAC), plant extracts, or carbomer impacted the 3-month activity of the samples differently. Important differences existing in the activities of ET and IPA, as a function of formulation factors or use conditions have been quantified using in vitro methods. Formulations should best be tailored for particular purposes and the all-purpose hand sanitizer may not exist.

**Supplementary Information:**

The online version contains supplementary material available at 10.1186/s41120-021-00038-x.

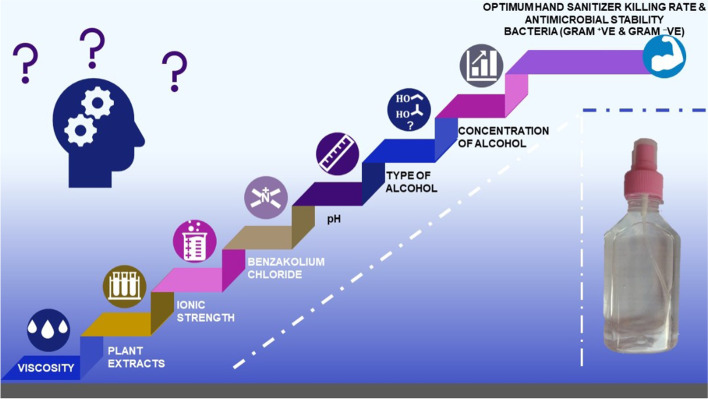

## Introduction

Hand hygiene utilizes handwashing or hand sanitizers to render the hands free of pathogens and has become more emphasized in the COVID-19 pandemic (Busari et al. 2012; World Health Organization 2020). A person’s contaminated hands can introduce harmful organisms to other body parts (Nicas and Best 2008). A recent study found that people touch their faces about 23 times an hour (Kwok et al. 2015). Contaminated hands have been reported as having the potential to spread respiratory infections (such as influenza and coronavirus) (Kwok et al. 2015; American Society for Microbiology 2003), with *Staphylococcus aureus* carried in the nasal mucosa in approximately 25% of the community (Wertheim et al. 2005; Munckhof et al. 2009), and may be acquired in this mode by individuals who are frequently exposed to potential carriers (Dancer 2008; Rongpharpi et al. 2013).

Nosocomial and community-acquired infectious have been linked to infectious particles transmissible through the hands (Allegranzi 2009). The use of effective hand rubs especially alcohol-based hand sanitizers (ABHS) is more than ever necessary to prevent the transmission of infectious particles both in the community and hospital environments, at the same time limiting the spread of diseases (Tusabe et al. 2020).

Alcoholic preparations are the main rinse-free sanitizers in the market, and they exert their actions by denaturing microbial proteins and inactivating viruses (Jing et al. 2020; Boyce and Pittet 2002). The biocidal activity of aliphatic alcohols, notably ethanol (ET) and isopropyl alcohol (IPA), has been observed at concentrations between 50 to 90 % against vegetative forms of specific bacteria and viruses including HIV (Hugo and Russell 2011). The presence of water is essential for activity. This is because it allows for adequate contact between the alcohol and important cellular components (Hugo and Russell 2011). Thus, such diluted alcohol efficiently penetrates the bacterial cell wall and achieves a rapid biocidal activity. Poor hand hygiene with emphasis on lack of compliance with disinfection procedures both at the hospital and in the community is perceived as the major factor that contributes to the transmission of infection (Hugo and Russell 2011). The formation and persistence of biofilms on surfaces as well as the transmission of infectious viral particles such as coronavirus 2 (SARS-CoV-2) contained in droplets on contaminated surfaces call for more stringent measures aimed at ensuring adequate use of disinfectants in addition to adherence to disinfection policies (Kampf et al. 2020). Thus, the World Health Organization (WHO) recommends that alcohol-based hand sanitizers contain at least 60% alcohol (World Health Organization 2020), as the alcohol concentration is a critical factor in determining if a sanitizer is biocidal or biostatic (Thaddeus et al. 2018). This is in line with literature information on the bioactivity of ABHS, supporting a rapid killing rate as well as a broad spectrum of activity against bacteria and viruses through protein denaturation when used at 62–95% (Jing et al. 2020). Also, when alcohols are used at a concentration of 85%, the available water for activity (avw) ensures the entrapment of the alcohol while it exhibits its action on the cell wall of the microbe.

The antimicrobial property of any formulation is affected by certain physicochemical properties, such as pH, electrolyte concentration, presence of organic matter, and the type of active ingredient, in this case ethyl alcohol or isopropyl alcohol.

The hypothesis is that formulation conditions/additives affect the disinfection activity of the two alcohols differently. S*taphylococcus aureus and Escherichia coli* are used as model Gram-positive and Gram-negative organisms respectively. Thus, this present study aimed at evaluating the effects of various formulation conditions and additives on the microbial killing rates of both freshly prepared and aged ethyl alcohol and isopropyl alcohol-based hand sanitizers against the above selected organisms.

## Methods

### Materials

ET (Sigma-Aldrich) 99.5%, IPA (Sigma-Aldrich) 99.5%, benzalkonium chloride (LOBA Chemie, India) 99.8%, sodium chloride (SAFC) 99.5%, sodium hydroxide pellet (Sigma-Aldrich) 98%, hydrochloric acid (Supelco) 99.9%, Mueller Hinton agar (Oxoid Limited, England), *Eschericheria coli* (*E. coli*) CP026939.1, and *Staphylococcus aureus* (*S. aureus)* CP0486331.3 from laboratory stocks kept in the Department of Pharmaceutical Microbiology and Biotechnology of our Institution. Plant parts were locally procured.

## Methods

### Antibacterial activities of freshly prepared solutions of ET and IPA

#### Comparison of the antibacterial activities without additives

ET and IPA both of 85% concentration were prepared by appropriately diluting the commercial alcohols using distilled water, as previously reported (Thaddeus et al. 2018). To test with the organisms, a tube containing a sample of the sanitizer was placed in a water bath regulated at between 45 and 50°C. At this equilibrium temperature, it was inoculated with 0.1mL of each of the organisms (*S. aureus* and *E. coli*), to achieve a concentration of approximately 10^6^ CFU/mL. At selected time points (15, 30, 45, and 60 s, excluding zero), aliquots were removed from each reaction mixture and plated onto the surface of a corresponding sterile nutrient agar held in a petri dish. The dish was then incubated at 37^°^C for 18 to 24 h. A negative control similarly inoculated but having no agent was prepared and incubated. Colonies were enumerated and reductions in viable counts were calculated for each formulation.

#### Influence of pH

A pH meter (JENWAY) was calibrated before use by determining the pH readings of buffer solutions of known pH (buffer 4 and 9). By adding either 0.1 N hydrochloric acid or 0.1 N sodium hydroxide solution, the pH was varied to attain final pH values of 1, 4, 5, 6, 7, and 9 for each alcohol. The killing rates of the formulations were then evaluated as before.

#### Influence of electrolyte concentration

Three concentrations (0.05 N, 0.1 N, and 0.2 N) of sodium chloride (NaCl) were prepared using distilled water. These solutions were used as vehicles to dilute the commercial alcohol to form 85 v/v solution of each alcohol in saline. The antimicrobial activity of each formulation was evaluated as above.

#### Influence of additives (herbal extract and benzalkonium chloride)

Carrot, cucumber, and aloe vera (200 g of each) were blended separately with 500 ml of distilled water, and the mixture was filtered using a sieve. The filtrates were collected and separately used to dilute the commercial alcohols as before. A stock solution of benzalkonium chloride (0.05%) was prepared and similarly used to dilute the alcohols. The killing rates of the formulations were then evaluated as before.

#### Influence of carbomer concentration

The calculated weight of the carbomer was sprinkled on the surface of a small volume (40 ml) of water, stirring for about 10 min. The homogenous dispersion was placed in a bottle and corked for about 2 days to hydrate. At the end of the period, 62.5 mL of 85% alcohol was added. The pH was noted, and then triethanolamine was introduced in drops. The neutralization was stopped when a final pH in the range of 6.2 to 6.5 was reached. The gel was then made up to 100 ml with the alcoholic solution.

### Antibacterial activities of solutions of ET and IPA after 3 months

The killing rates of all preparation tested before were re-evaluated after 3 months of storage at room temperature (25 to 30°C).

### Statistical analysis

Two-way ANOVA with Bonferroni post-tests for multiple comparisons was used to analyze the effect of each parameter. All graphs and statistical analyses were made using GraphPad Prism 5.0. Statistical significance was tested at 95% confidence interval. Where appropriate, microbial death being the dependent variable has been presented as mean ± standard deviation.

## Results

### Killing rates of freshly prepared solutions

#### Killing rates of the alcohols without additives

The microbial death (*S. aureus*) seen with ET (Table 1) in 15 s (98.53%) is lower than with IPA (100%) (*P* < 0.001). Against *E. coli*, ET achieved 100% killing effect within 15s of treatment, which was higher (*P* = 0.018) than the value for IPA (99.77%) in the same interval of exposure. From a two-way ANOVA, both the time of exposure and the sanitizer type affected the activity against *S. aureus*, whereas for *E. coli*, time of exposure is significant while sanitize type is less significant*.*

**Table 1:** Killing rates of 85% ethanol and 85% isopropyl alcohol against *Staphylococcus aureus* and *Escherichia coli*

Time	***S. aureus***		***E. coli***	
	Ethanol	Isopropyl alcohol	Ethanol	Isopropyl alcohol
15	98.53 ± 0.00	100.00 ± 0.00*	100.00 ± 0.00	99.77 ± 0.20*
30	99.14 ± 0.21^**†**^	100.00 ± 0.00*	100.00 ± 0.00	100.00 ± 0.00^**†**^
45	99.02 ± 0.21^**†**^	100.00 ± 0.00b*	100.00 ± 0.00	100.00 ± 0.00^**†**^
60	99.26 ± 0.00^**†**^	100.00 ± 0.00*	100.00 ± 0.00	100.00 ± 0.00^**†**^

Values represent means ± SD. Values with * indicate a significant difference (*P* < 0.05) from ethanol for the same time/organism. Values with superscript † indicate a significant difference (*P* < 0.05) from 15 s for the same alcohol/organism. *N* = 3

#### Effects of pH adjustment on killing rates of freshly prepared solutions

**Fig. 1:**
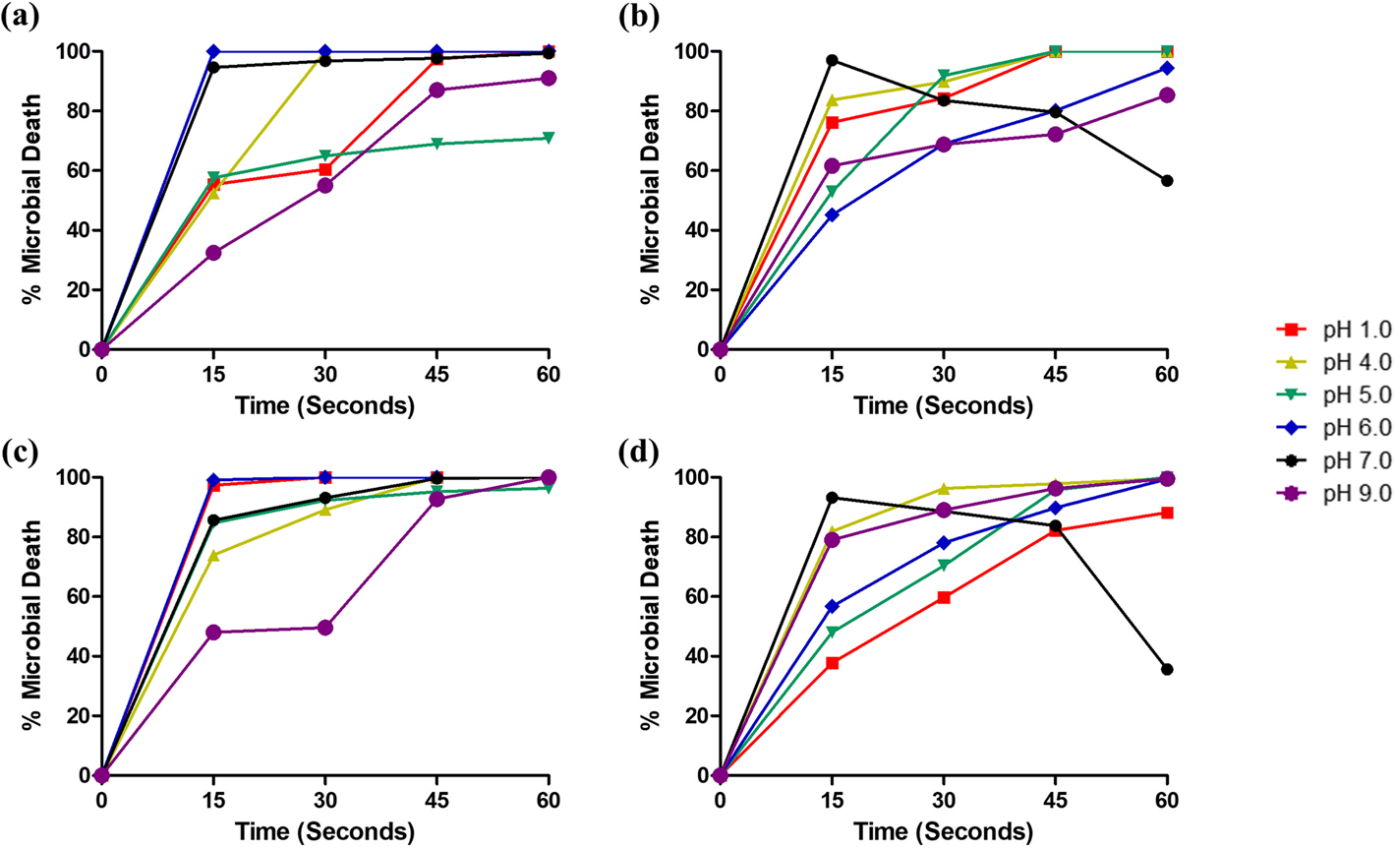
Effect of varying the pH on the percentage microbial death (mean ± S.D.) recorded by freshly prepared alcoholic solutions **a** 85% ethanol, *S. aureus* (Et); **b** 85% isopropyl alcohol (IPA), *S. aureus*; **c** 85% ethanol (ET), *E. coli*; and **d** 85% isopropyl alcohol (IPA), *E. coli. N* = 3

Ethanol exhibited maximum activities around pH 6, with 100% ± 0.00 microbial death against *S. aureus* (Fig. 1a) and 99.09% ± 0.52 microbial death against *E. coli* (Fig. 1c) in 15s (Fig. 1a). Microbial deaths were lower for other pHs. For IPA (Fig. 1b, d), transient peak activity was observed in 15 s at pH 7 for both *S. aureus* (97.06 ± 0.74, Fig. 1b) and *E. coli* (93.20 ± 0.68, Fig. 1d). A subsequent fall was observed after this time.

#### Effects of electrolyte concentration on killing rates of freshly prepared solutions

**Fig. 2:**
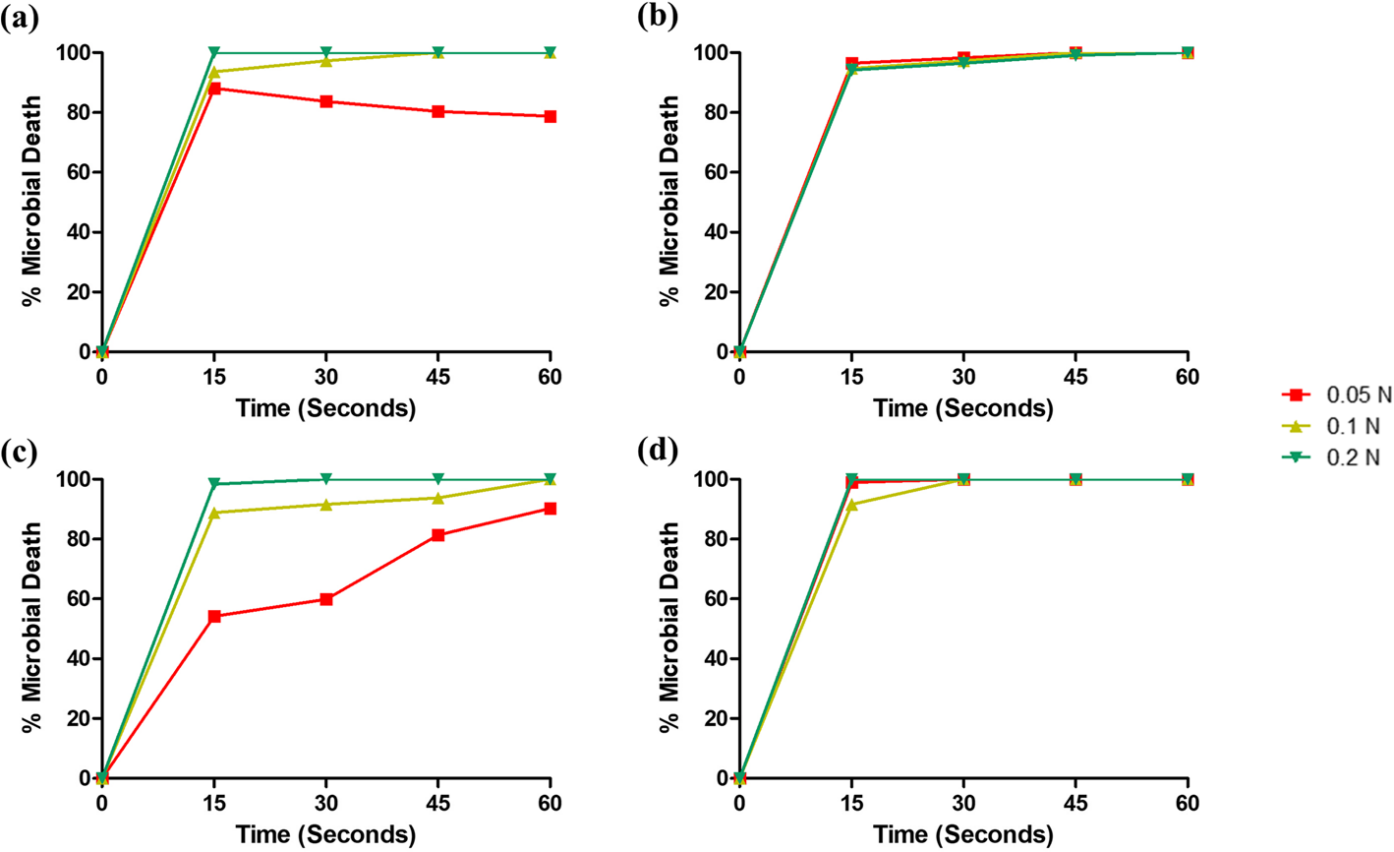
The effects of varying electrolyte concentration on the percentage microbial death (mean ± S.D.) recorded by freshly prepared alcoholic solutions **a** 85% ethanol (ET), *S. aureus*; **b** 85% isopropyl alcohol (IPA), *S. aureus*; **c** 85% ethanol (ET), *E. coli*; and **d** 85% isopropyl alcohol (IPA), *E. coli*. *N* =3

Ethanol combined with high electrolyte concentration (Fig. 2a, c) achieved a faster killing rate against both organisms. With IPA (Fig. 2b, d), variations in antimicrobial activity with electrolyte concentration were less pronounced for both organisms.

#### Effects of different additives on killing rates of freshly prepared solutions

**Fig. 3:**
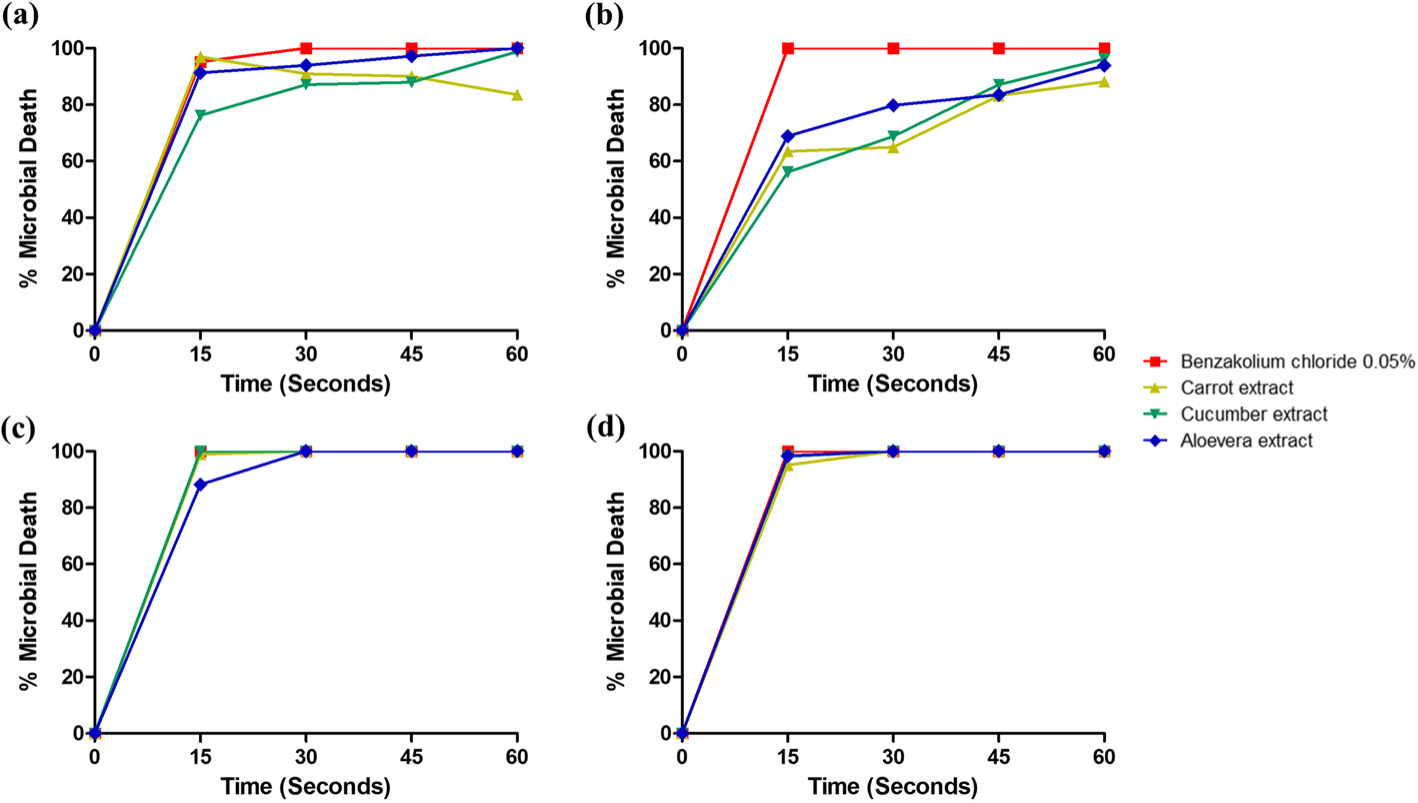
The effects of the presence of different additives on the percentage microbial death (mean ± S.D.) recorded by freshly prepared alcoholic solutions **a** 85% ethanol (ET), *S. aureus*; **b** 85% isopropyl alcohol (IPA), *S. aureus*; **c** 85% ethanol (ET), *E. coli*; and **d** 85% isopropyl alcohol (IPA), *E. coli. N* =3

The addition of a low concentration of benzalkonium chloride tended to enhance the activity of IPA (Fig. 3b, d) against the two organisms more than ET (Fig. 3a, c). The presence of extracts negatively impacted the ability of both ET and IPA to kill *S. aureus* with a more pronounced effect on IPA (Fig. 3b). Of all the mixtures tested against *S. aureus*, none achieved 100% microbial death before 60 s. With *E. coli*, *only* ET-cucumber extract achieved 100% microbial death in 15 s.

#### Effects of carbomer on killing rate of freshly prepared solutions

**Fig. 4:**
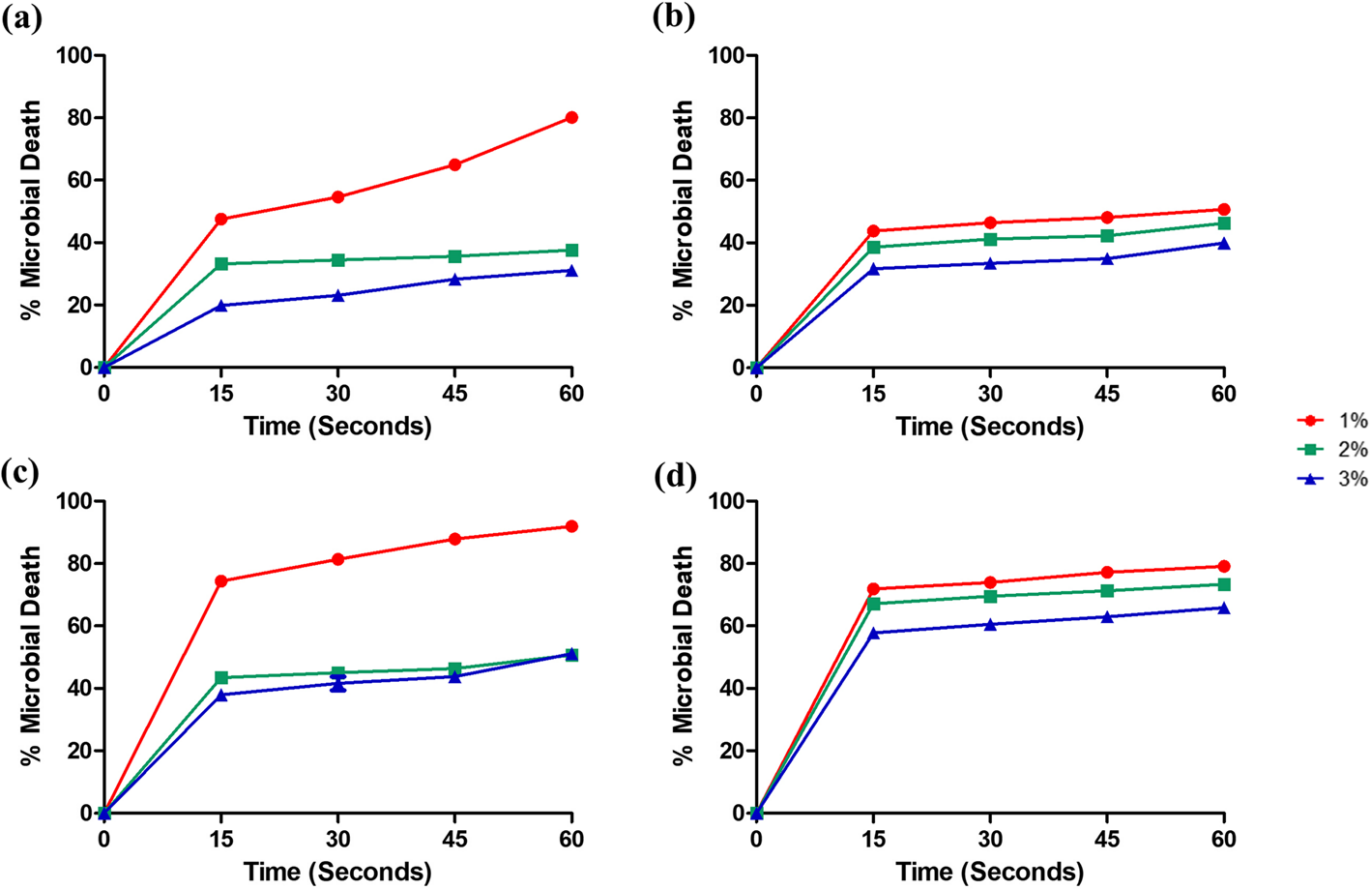
The effects of carbomer concentration on the percentage microbial death (mean ± S.D.) recorded by freshly prepared alcoholic solutions **a** 85% ethanol (ET), *S. aureus*; **b** 85% isopropyl alcohol (IPA), *S. aureus*; **c** 85% ethanol (ET), *E. coli*; and **d** 85% isopropyl alcohol (IPA), *E. coli*. *N* = 3

For both alcohols, the percentage microbial death decreased with increasing carbomer concentration (Fig. 4a–d). With the addition of a carbomer, none could achieve 100% bacterial death in 60 s.

### Killing rates of alcohols after 3 months

#### Effects of pH adjustment on 3-month killing rate

At pH 6, the 15 s- activity of ET against *S. aureus* was maintained even after 3 months of preparation as no reduction was found from the 100% microbial death of the freshly prepared sample. (Table 2). However, its 15 s activity against *E. coli* was reduced (from 99.09 to 94.90%). On storage, the 15 s activity of the solution adjusted to pH 7 (where IPA freshly prepared IPA showed maximum 15 s activity) against *S. aureus* reduced, whereas the activity against *E. coli* did not. A comparison of the influence of pH on the 60 s activities of the two alcohols on storage is presented in Additional file 1: Appendix A.

**Table 2:** Three-month assessment of microbial death at 15 s for alcohol solutions at different pHs

pH	Alcohol	Microbial death (%)			
		*S. aureus*		*E. coli*	
		Fresh	3 months	Fresh	3 months
1.0	Ethanol	55.39 ± 1.29	8.83 ± 0.97*	97.39 ± 0.52	95.92 ± 0.90
	Isopropyl alcohol	76.23 ± 0.93^**†**^	59.68 ± 2.02*^†^	37.75 ± 0.91^**†**^	−3.97 ± 1.74*^†^
4.0	Ethanol	52.45 ± 0.93	7.23 ± 0.93*	73.92 ± 0.71	62.25 ± 1.23*
	Isopropyl alcohol	83.70 ± 1.18^**†**^	65.93 ± 0.93*^†^	81.86 ± 1.19^**†**^	75.51 ± 0.68*^†^
5.0	Ethanol	57.60 ± 1.66	7.23 ± 0.93*	84.69 ± 0.68	76.98 ± 0.86*
	Isopropyl alcohol	52.94 ± 1.10^**†**^	27.33 ± 0.93*^†^	48.07 ± 0.52^**†**^	17.01 ± 4.14*^†^
6.0	Ethanol	100.00 ± 0.00	98.28 ± 0.57	99.09 ± 0.52	94.90 ± 0.68*
	Isopropyl alcohol	45.10 ± 0.93^**†**^	28.18 ± 2.09*^†^	56.69 ± 0.86^**†**^	14.97 ± 0.68*^†^
7.0	Ethanol	94.61 ± 0.93	88.11 ± 0.93*	85.60 ± 0.86	77.89 ± 1.02*
	Isopropyl alcohol	97.06 ± 0.74	92.04 ± 0.76*^†^	93.20 ± 0.68^**†**^	90.03 ± 1.37^**†**^
9.0	Ethanol	32.47 ± 1.29	7.23 ± 0.93*	48.07 ± 1.04	2.04 ± 0.68*
	Isopropyl alcohol	61.64 ± 1.66^**†**^	24.51 ± 3.08*^†^	79.02 ± 0.71^**†**^	67.69 ± 0.68*^†^

Values represent means ± SD. Values with * indicate a significant difference (*P* < 0.05) from fresh sample for the same alcohol/pH/organism. Values with ^**†**^ indicate a significant difference (*P* < 0.05) from ethanol for the same pH/organism/test time. *N* = 3

#### Effects of electrolyte concentration on 3-month killing rate

On storage for 3 months, ET maintained its activity against both *S. aureus* and *E. coli* at high electrolyte concentration (0.20 N) (Table 3). With IPA, on storage, the 15-s activities against both organisms were reduced for all electrolyte concentrations*.* A comparison of the influence of electrolyte concentration on the 60 s activities of the two alcohols on storage is presented in Additional file 1: Appendix B.

**Table 3:** Three-month assessment of microbial death at 15 s for alcohol solutions at different electrolyte concentrations

NaCl conc. (*N*)	Alcohol	Microbial death (%)			
		*S. aureus*		*E. coli*	
		Fresh	3 months	Fresh	3 months
0.05	Ethanol	88.11 ± 0.93	83.33 ± 0.93*	54.19 ± 0.86	31.06 ± 2.19*
	Isopropyl alcohol	96.45 ± 0.93^**†**^	93.87 ± 0.57*^†^	98.98 ± 0.34a^**†**^	95.69 ± 0.86*^†^
0.10	Ethanol	93.63 ± 0.77	90.81 ± 0.64*	88.89 ± 0.71	75.62 ± 0.86*
	Isopropyl alcohol	94.73 ± 1.12	92.28 ± 0.74*	91.61 ± 0.86a^**†**^	88.09 ± 1.80*^†^
0.20	Ethanol	100.00 ± 0.00	98.65 ± 0.56	98.41 ± 0.52	96.49 ± 0.52
	Isopropyl alcohol	94.24 ± 0.56^**†**^	87.26 ± 0.93*^†^	100.00 ± 0.00^**†**^	96.94 ± 0.68*

Values represent means ± SD. Values with * indicate a significant difference (*P* < 0.05) from fresh sample for the same alcohol/electrolyte/organism. Values ^**†**^ indicate significant difference (*P* < 0.05) from ethanol for the same electrolyte/organism/test time. *N* = 3

#### Effects of additives on 3-month killing rate

The activity of IPA-BAC against both bacteria was maintained in the 3-month interval (Table 4). The activity of ET-BAC was reduced for both organisms in the test period. Only the ET-carrot mixture maintained its activity against both bacteria after the time interval. The activities of ET-cucumber and IPA-aloe vera were maintained against *E. coli* only. A comparison of the influence of additives on the 60 s activities of the alcohols on storage is presented in Additional file 1: Appendix C.

**Table 4:** Three-month assessment of microbial death at 15 s for alcohol solutions containing different additives

Additives	Alcohol	Microbial death (%)			
		*S. aureus*		*E. coli*	
		Fresh	3 months	Fresh	3 months
Benzalkonium chloride (0.05%)	Ethanol	95.22 ± 0.74	90.81 ± 1.32*	100.00 ± 0.00	96.49 ± 0.71*
	Isopropyl alcohol	100.00 ± 0.00^**†**^	97.67 ± 0.56	100.00 ± 0.00	98.98 ± 0.68^**†**^
Carrot extract	Ethanol	96.94 ± 0.92	96.94 ± 1.53	98.98 ± 0.34	98.30 ± 0.34
	Isopropyl alcohol	63.48 ± 0.76^**†**^	51.96 ± 1.12*	95.13 ± 0.86^**†**^	90.59 ± 0.86*^†^
Cucumber extract	Ethanol	76.22 ± 0.76	70.71 ± 0.56*	100.00 ± 0.00	98.98 ± 0.68
	Isopropyl alcohol	56.13 ± 0.57^**†**^	23.41 ± 2.81*	98.19 ± 0.52^**†**^	93.77 ± 0.52*^†^
Aloe vera extract	Ethanol	91.30 ± 1.12	84.07 ± 0.77*	88.21 ± 0.86	86.17 ± 1.75*
	Isopropyl alcohol	68.87 ± 0.93^**†**^	58.33 ± 1.53*	98.41 ± 0.52^**†**^	97.28 ± 0.68^**†**^

Values represent means ± SD. Values with * indicate a significant difference (*P* < 0.05) from fresh sample for the same alcohol/additive/organism. Values with ^**†**^ indicate significant difference (*P* < 0.05) from ethanol for the same additive/organism/test time. *N* = 3

#### Effects of carbomer concentration on a 3-month killing rate

As shown in Table 5, the activities of the alcohol-carbomer mixtures generally declined after 3 months except with ET-carbomer which exhibited significantly increased activity at 3% carbomer concentration for both organisms. A comparison of the influence of carbomer on the 60 s activities of the alcohols on storage is presented in Additional file 1: Appendix D.

**Table 5:** Three-month assessment of microbial death at 15 s for alcohol solutions containing different carbomer concentrations

Carbomer conc. (%)	Alcohol	Microbial death (%)			
		*S. aureus*		*E. coli*	
		Fresh	3 months	Fresh	3 months
1	Ethanol	47.55 ± 0.93	32.84 ± 0.92*	74.38 ± 0.86	67.80 ± 0.52*
	Isopropyl alcohol	43.75 ± 0.74^**†**^	39.46 ± 0.93*^†^	71.88 ± 0.71^**†**^	63.16 ± 0.52*^†^
2	Ethanol	33.21 ± 0.77	29.04 ± 1.32*	43.43 ± 1.04	57.93 ± 1.19*
	Isopropyl alcohol	38.60 ± 0.74^**†**^	36.27 ± 0.93*^†^	67.12 ± 0.52^**†**^	57.03 ± 0.71*
3	Ethanol	19.85 ± 1.11	25.25 ± 2.03*	37.98 ± 0.86	47.05 ± 0.98*
	Isopropyl alcohol	31.74 ± 0.56^**†**^	30.88 ± 0.98^**†**^	57.82 ± 1.23^**†**^	52.95 ± 0.52*^†^

Values represent means ± SD. Values with * indicate a significant difference (*P* < 0.05) from fresh sample for the same alcohol/carbomer/organism. Values with ^**†**^ indicate a significant difference (*P* < 0.05) from ethanol for the same test time/carbomer/organism. *N* = 3

## Discussion

ET and IPA are known to be rapidly germicidal against vegetative bacteria, fungi, viruses, and even tuberculosis cells (Rutala and Weber 2008). Even though IPA had been reported to be slightly more active than ET against both *S. aureus* and *E. coli (*Rutala and Weber 2008*;* Coulthard and Sykes 1936*)*, our results suggest that ET is slightly more active against *E. coli* whereas IPA is more active against *S. aureus*.

An ideal sanitizer would be formulated to conditions that guarantee the elimination of 100% of viable cells in a very short time interval. This is because the occurrence of residual organisms can have disastrous consequences under certain conditions (Nye and Mallory 1923). In a previous report using the agar diffusion method (Thaddeus et al. 2018), it was demonstrated using a number of organisms that the activity of aqueous alcohols peaked at around 85% v/v, with 95 v/v shown to have reduced activity in dry environments (Morton 1950).

The two alcohols form slightly basic solutions in the water of pH in the range of about 7.5 to about 8.5. Even though alcohols have been shown to be germicidal at concentrations lower than 50%, or even as low as 20%, (Rutala and Weber 2008), pH would still play a crucial role at any concentration applied. For results obtained with 85% v/v alcohol, IPA is very sensitive to variations in pH (more than ET). Therefore, in the disinfection of any material whose surface pH differs from the pH of the formulation itself, such as alkaline surfaces and surgical materials, the pH of the material must be factored in. This consideration also becomes important in the storage of sanitizer solutions in glass containers which may leach alkaline agents into the alcoholic solution, thereby tilting the solution pH towards neutral or the alkaline ranges. For this reason, containers for alcoholic preparations including sanitizers and methylated spirits should be carefully selected.

It has been demonstrated that acidification of ET can help in the rapid destruction of bacterial spores of *Clostridium officinale* (Nerandzic et al. 2015), implying that alcoholic disinfectants may serve limited roles as sterilants with controlled pH modification. This would need to be investigated further with other spore formers. However, as shown with both *E.coli* and *Staph aureus*, only a slightly acid pH would be needed for ET. From Table 1, the microbial death at 15 s is statistically different from values for 30, 45, and 60 s, so *S. aureus* would need to be exposed to ET for up to 30 s in order to guarantee complete elimination. For IPA, there is no statistical difference between the killing rate at 15 s and the other times, and this suggests that IPA acts faster than ET against *S. aureus*. For *E. coli*, the microbial death at 15 s following exposure to ET is statistically insignificant from values for higher exposure times, whereas for IPA the values are significant. Therefore, for the alcohols, more than 15 s, preferably up to 30 s of rubbing time would be better since the microbial contaminants on the hands would most probably consist of both G-ve and G+ve. The authors are aware that the results presented above may be influenced not just by the pH (hydrogen ion concentration), but also by the presence of sodium (Na^+^) and chloride (Cl^-^) ions introduced in the course of pH adjustment.

At the three electrolyte concentrations tested, there were wider effects on the activity of ET than with IPA. It had been previously demonstrated that a high electrolyte concentration was important in enhancing the activity of ET against bacterial spores (Nerandzic et al. 2015). Additionally, high electrolyte concentration may potentially salt out IPA from the solution, since it is not miscible with salt solutions (Zhigang et al. 2001). The results indicate that ET is more stable in higher electrolyte concentration than IPA because the killing rates under this condition are not different between 0 and 3 months for the two organisms at 15-s test time.

With the inclusion of plant extracts, wider variations were seen in the activities of the alcohols against *S. aureus* than with *E. coli*. The common drivers of brand acceptance include sensory attributes such as viscosity and emollience, the degree of the “cold sensation” produced on application and rubbing, and the presence of fragrance. The inclusion of aloe vera gel in commercial hand sanitizers aims to improve fibroblast activity leading to collagen synthesis while also conferring some protection from harmful ultraviolet radiation (Surjushe et al. 2008; Heggers et al. 1996; Chithra et al. 1998). On the other hand, cucumber is believed to moisturize the skin, inhibit melanin synthesis, and to have anti-inflammatory, anti-irritant, anti-wrinkling, and skin-brightening effects (Akhtar et al. 2011; Murad and Nyc 2012; Hooda 2015). Carrot is the ideal vitaminized food and several of its constituents such as ascorbic acid and beta-carotene, in addition to phenolic compounds, can help in maintaining the integrity of the skin and connective tissues (Sharma et al. 2012). Vitamin C and beta carotene especially would be expected to be beneficial in healing and regeneration of the skin of the hand.

Not minding the benefits outlined above, these extracts should be used sparingly in hand sanitizer formulations, since they can exert subtle changes in the killing rate in the short time of application of a hand sanitizer. They are also more suitable for leave-on skincare products where dirt build-up may not be a problem.

In a previous report (Thaddeus et al. 2018), it was demonstrated that benzalkonium chloride (BAC) potentiated the activity of IPA (both inhibition zone diameter and spectrum) in a concentration-dependent manner. Many proprietary products contain as high as 1% of benzalkonium chloride. By adding BAC to alcohol, it may be possible to achieve both immediate and long-lasting actions while using lower concentrations of the quaternary ammonium compound (QAC). When used alone in high concentrations, QACs are known to pose a threat to health, especially with repeated use. Due to low volatility, they can also linger on surfaces for considerable periods after application. The use of alcohol reinforced with BAC may be more suitable for increasing the spectrum of activity, such as during surface cleaning and disinfection, and also for achieving both immediate and long-lasting effects. It may also be used for hand disinfection where heavy contamination with different organisms is suspected. Of all the additives, the activity of the IPA-BAC mixture did not decline after 3 months of storage. BAC therefore can be combined with IPA-based sanitizers. Moreover, it was previously noted to improve the overall spectrum of activity of IPA (Thaddeus et al. 2018). Its inclusion also seems to affect the 3-month killing rate of ET more than IPA. For IPA, the killing rates at the two times were not different for both organisms.

This study was informed in part by an observation that users of hand sanitizers seemed to have a preference for very viscous preparations and those that have been formulated with gel-forming plant extracts such as aloe vera. Carbomer imparts viscosity and this can positively reduce the volatility of the alcohol and allow for adequate contact time during which most vegetative organisms can be neutralized. However, such high concentrations can also result in the build-up of residual films of thickener on the hands, making further hand disinfection with alcohols difficult without handwashing. High carbomer concentration is more tolerated by ET than IPA, as shown in Table 5 (with 3% carbomer).

One limitation of this study is the fact that the killing rate was tested under non-rubbing conditions (without friction). From the results, however, it is evident that alcohol-based hand sanitizers should be as minimal in terms of composition as possible. Lighter compositions are also more suitable for use on a variety of surfaces, such as office front desks where thick preparations will leave residues. The problem here is that alcohol-only products have limited cosmetic appeal. Reduced antimicrobial action due to glycerol had been previously reported (Thaddeus et al. 2018; Suchomel et al. 2013; Menegueti et al. 2019). The principal driver of demand should be the alcohol content, not the presence of any other ingredient. In the COVID-19 pandemic, alcohol-free sanitizers are not recommended for hand hygiene (Berardi et al. 2020). The formulation as a thick gel especially reduces the disinfection rate or increases the disinfection time (Kramer et al. 2002).

With most chemical vendors, IPA is approximately 1.5 times more expensive than ET for a 220-l container. This is expected to negatively impact the price of unit packs of hand sanitizers formulated with IPA. One of the mainstays of the anti-COVID19 efforts is regular disinfection of the hands using hand sanitizers, which is often a more feasible alternative to handwashing with soap and water. Though viruses were beyond the scope of this study, ET has been reported to have a wider spectrum than IPA against viruses, being fully active against all lipid-enveloped viruses (herpes and influenza inclusive) and many hydrophilic viruses (adenoviruses, rotaviruses, rhinoviruses, and enteroviruses, as well as the human immunodeficiency virus (HIV) (Martin et al. 1985). IPA has no activity against hydrophilic viruses. Besides formulating in the optimum concentration range (Thaddeus et al. 2018; Morton 1950; Ali et al. 2001), sanitizers should be as lean as possible in terms of additives.

## Conclusion

From the in vitro test results, the two alcohols exhibited different antimicrobial killing rates when formulation conditions or composition was varied. Clinicians and other health workers should be aware of this in recommending hand sanitizers since a variety of factors (pH of the hand/surface, presence of dirt, type, and degree of contamination) affect the antimicrobial performance and can make it impossible to achieve the same rate and or level of disinfection. A single disinfection failure can be very expensive and in the COVID-19 pandemic, safe, and reliable hand sanitization will assume far greater importance than before. In the future, hand sanitizers may be formulated for specific conditions or organisms by employing formulation conditions that have been specifically optimized for them.

## Supplementary Information

**Additional file 1.**

## Data Availability

All data generated or analyzed during this study are included in this published article [and its supplementary information files].
