# Supplementary material for: Designing an ideal alcohol-based hand sanitizer: in vitro antibacterial responses of ethanol and isopropyl alcohol solutions to changing composition
Source: AAPS Open. 2021 Nov 22;7(1):5. doi: 10.1186/s41120-021-00038-x (PMC8606244; doi:10.1186/s41120-021-00038-x)
Supplement: Supplementary file 1 — Additional file 1. [file 41120_2021_38_MOESM1_ESM.docx]

Appendix I: The microbial death at 60 seconds for alcohol solutions at different pHs

| pH | Alcohol | Microbial Death (%) | | | | |
| --- | --- | --- | --- | --- | --- | --- |
|  |  | *S. aureus* | |  | *E. coli* | |
|  |  | Fresh | 3 Months |  | Fresh | 3 Months |
| 1.0 | Ethanol | 100.00 ± 0.00 | 98.77 ± 0.56 |  | 100.00 ± 0.00 | 100.00 ± 0.00 |
|  | Isopropyl alcohol | 100.00 ± 0.00 | 100.00 ± 0.00 |  | 88.21 ± 0.86**^†^** | 84.92 ± 1.04***^†^** |
| 4.0 | Ethanol | 100.00 ± 0.00 | 100.00 ± 0.00 |  | 100.00 ± 0.00 | 99.43 ± 0.52 |
|  | Isopropyl alcohol | 100.00 ± 0.00 | 95.10 ± 0.93***^†^** |  | 99.66 ± 0.59 | 95.92 ± 0.90***^†^** |
| 5.0 | Ethanol | 70.83 ± 0.93 | 50.25 ± 1.29* |  | 96.37 ± 0.52 | 94.11 ± 0.86* |
|  | Isopropyl alcohol | 100.00 ± 0.00**^†^** | 98.41 ± 0.57**^†^** |  | 100.00 ± 0.00**^†^** | 98.87 ± 0.52**^†^** |
| 6.0 | Ethanol | 100.00 ± 0.00 | 100.00 ± 0.00 |  | 100.00 ± 0.00 | 100.00 ± 0.00 |
|  | Isopropyl alcohol | 94.49 ± 0.74**^†^** | 91.54 ± 0.74***^†^** |  | 99.43 ± 0.39 | 94.90 ± 0.90***^†^** |
| 7.0 | Ethanol | 99.39 ± 0.77 | 95.43 ± 0.43* |  | 100.00 ± 0.00 | 100.00 ± 0.00 |
|  | Isopropyl alcohol | 56.62 ± 1.94**^†^** | 29.41 ± 1.10***^†^** |  | 35.60 ± 1.04**^†^** | 27.78 ± 0.86***^†^** |
| 9.0 | Ethanol | 91.05 ± 1.13 | 87.38 ± 0.77* |  | 100.00 ± 0.00 | 99.09 ± 0.52 |
|  | Isopropyl alcohol | 85.42 ± 0.56**^†^** | 76.35 ± 1.18***^†^** |  | 99.55 ± 0.79 | 95.24 ± 0.68***^†^** |

Appendix I legend: Values represent Means ± SD. Values with * indicate significant difference (p < 0.05) from fresh sample for the same alcohol/organism/pH. Values with **^†^**  indicate significant difference (p < 0.05) from ethanol for the same pH/organism/test time.

Appendix II: The microbial death at 60 seconds for alcohol solutions at different electrolyte concentration

| Electrolyte concentration  (N) | Alcohols | Microbial Death (%) | | | | |
| --- | --- | --- | --- | --- | --- | --- |
|  |  | *S. aureus* | |  | *E. coli* | |
|  |  | Fresh | 3 Months |  | Fresh | 3 Months |
| 0.05 | Ethanol | 78.8 ± 0.93 | 71.20 ± 077* |  | 90.25 ± 0.52 | 84.80 ± 0.52* |
|  | Isopropyl alcohol | 100.00 ± 0.00**^†^** | 100.00 ± 0.00**^†^** |  | 100.00 ± 0.00**^†^** | 100.00 ± 0.00**^†^** |
| 0.10 | Ethanol | 100.00 ± 0.00 | 100.00 ± 0.00 |  | 100.00 ± 0.00 | 100.00 ± 0.00 |
|  | Isopropyl alcohol | 100.00 ± 0.00 | 100.00 ± 0.00 |  | 100.00 ± 0.00 | 100.00 ± 0.00 |
| 0.20 | Ethanol | 100.00 ± 0.00 | 100.00 ± 0.00 |  | 100.00 ± 0.00 | 100.00 ± 0.00 |
|  | Isopropyl alcohol | 100.00 ± 0.00 | 93.50 ± 0.57***^†^** |  | 100.00 ± 0.00 | 100.00 ± 0.00 |

Appendix II legend: Values represent Means ± SD. Values with * indicate significant difference (p < 0.05) from fresh sample for the same alcohol/electrolyte/organism. Values with **^†^** indicate significant difference (p < 0.05) from ethanol for the same electrolyte/organism/test time.

Appendix III: The microbial death at 60 seconds for alcohol solutions containing different additives

| Additives | Alcohol | Microbial Death (%) | | | | |
| --- | --- | --- | --- | --- | --- | --- |
|  |  | *S. aureus* | |  | *E. coli* | |
|  |  | Fresh | 3 Months |  | Fresh | 3 Months |
| Benzalkonium (0.05%) | Ethanol | 100.00 ± 0.00 | 100.00 ± 0.00 |  | 100.00 ± 0.00 | 100.00 ± 0.00 |
|  | Isopropyl alcohol | 100.00 ± 0.00 | 100.00 ± 0.00 |  | 100.00 ± 0.00 | 100.00 ± 0.00 |
| Carrot extract | Ethanol | 83.58 ± 0.93 | 69.85 ± 1.94* |  | 100.00 ± 0.00 | 100.00 ± 0.00 |
|  | Isopropyl alcohol | 88.11 ± 0.93**^†^** | 66.91 ± 2.57***^†^** |  | 100.00 ± 0.00 | 100.00 ± 0.00 |
| Cucumber extract | Ethanol | 98.77 ± 0.56 | 96.32 ± 0.37* |  | 100.00 ± 0.00 | 100.00 ± 0.00 |
|  | Isopropyl alcohol | 96.20 ± 0.77**^†^** | 91.91 ± 1.10***^†^** |  | 100.00 ± 0.00 | 100.00 ± 0.00 |
| Aloe vera extract | Ethanol | 100.00 ± 0.00 | 96.94 ± 0.57* |  | 100.00 ± 0.00 | 100.00 ± 0.00 |
|  | Isopropyl alcohol | 93.87 ± 0.57**^†^** | 87.99 ± 1.13***^†^** |  | 100.00 ± 0.00 | 100.00 ± 0.00 |

Appendix III legend: Values represent Means ± SD. Values with * indicate significant difference (p < 0.05) from fresh sample for the same alcohol/additive/organism Values with **^†^** indicate significant difference (p < 0.05) from ethanol for the same additive/organism/test time.

Appendix IV: The microbial death at 60 seconds for alcohol solutions at different carbomer concentrations

| Carbomer  Concentration(%) | Alcohol | Microbial Death (%) | | | | |
| --- | --- | --- | --- | --- | --- | --- |
|  |  | *S. aureus* | |  | *E. coli* | |
|  |  | Fresh | 3 Months |  | Fresh | 3 Months |
| 1 | Ethanol | 80.15 ± 1.11 | 57.97 ± 1.12* |  | 91.95 ± 1.04 | 88.55 ± 1.04* |
|  | Isopropyl alcohol | 50.74 ± 1.11**^†^** | 49.88 ± 1.49**^†^** |  | 79.14 ± 0.71**^†^** | 71.88 ± 0.86***^†^** |
| 2 | Ethanol | 37.62 ± 0.57 | 37.38 ± 0.57 |  | 50.68 ± 0.68 | 66.56 ± 0.52* |
|  | Isopropyl alcohol | 46.32 ± 1.11**^†^** | 41.42 ± 1.13***^†^** |  | 73.36 ± 0.86**^†^** | 64.74 ± 1.04***^†^** |
| 3 | Ethanol | 31.13 ± 0.93 | 35.17 ± 0.93* |  | 51.13 ± 1.04 | 54.65 ± 1.42* |
|  | Isopropyl alcohol | 39.95 ± 0.77**^†^** | 37.62 ± 0.57***^†^** |  | 65.88 ± 0.52**^†^** | 59.63 ± 0.52***^†^** |

Appendix IV legend: Values represent Means ±  SD. Values with * indicate significant difference (p < 0.05) from fresh sample for same alcohol/carbomer/organism Values with **^†^** indicate significant difference (p < 0.05) from ethanol for the same carbomer/organism/ test time.
